# Variegatusides: New Non-Sulphated Triterpene Glycosides from the Sea Cucumber *Stichopus variegates* Semper

**DOI:** 10.3390/md12042004

**Published:** 2014-04-02

**Authors:** Xiao-Hua Wang, Zheng-Rong Zou, Yang-Hua Yi, Hua Han, Ling Li, Min-Xiang Pan

**Affiliations:** 1 Research Center for Marine Drugs, School of Pharmacy, Second Military Medical University, 325 Guohe Road, Shanghai 200433, China; E-Mails: wangxiaohua737@sina.com (X.-H.W.); yiyanghua@hotmail.com (Y.-H.Y.); virgen2008@hotmail.com (M.-X.P.); 2 Department of Pharmacy, 202th Hospital of PLA, 5 Guangrong Street, Shenyang 110003, China; 3 College of Life Science, Jiangxi Normal University, 99 Ziyang Road, Nanchang 330022, China; E-Mail: zouzhr@163.com; 4 School of Medicine, Tongji University, 1239 Siping Road, Shanghai 200092, China; 5 Department of Pharmacy, 306th Hospital of PLA, 9 Anxiang North Road, Beijing 100101, China

**Keywords:** sea cucumber, *Stichopus variegates* Semper, triterpene glycoside, variegatuside, antifungal activity

## Abstract

Four new triterpene glycosides, variegatusides C–F (**1**–**4**), together with three structurally known triterpene glycosides, variegatusides A and B (**5**, **6**), and holothurin B (**7**), were isolated from the sea cucumber *Stichopus variegates* Semper (Holothuriidae), collected from the South China Sea. Their structures were elucidated on the basis of extensive spectral analysis (nuclear magnetic resonance (NMR) and electrospray ionization mass spectrometry (ESIMS)) and chemical evidence. Variegatusides C–F exhibit the same structural feature consisting of the presence of a 23-hydroxyl group at the holostane-type triterpene aglycone side chain. Variegatuside C (**1**) has a double bond (24, 25) in this same chain, while variegatuside D (**2**) exhibits a 8(9)-ene bond in the holostane-type triterpene aglycone, which has not been extracted from other sea cucumber species. Compound **4** is a native compound from the sea cucumber *S. variegates* Semper, which has been reported to be desacetylstichloroside B_1_. Except for holothurin B, these glycosides have no sulfate group in their sugar chain and show potent antifungal activities *in vitro* biotests.

## 1. Introduction

Triterpene glycosides are the predominant secondary metabolites of holothurians and are responsible for their general toxicity. These glycosides have been reported to have a wide spectrum of biological effects, including antifungal, cytotoxic, hemolytic, and immunomodulatory activities [1,2]. In the search for new pharmacologically active substances from marine organisms, attention has been paid to echinoderms, and among them, to sea cucumbers (class Holothuridea). More than 100 of these glycosides have been described, and the majority are usually lanosterol type triterpenes with an 18(20) lactone and a sugar chain of up to six monosaccharide units (princinally d-xylose, d-glucose, d-quinovose, d-3-*O*-methylglucose and d-3-*O*-methylxylose) linked to the C-3 of the aglycone [3]. As a continuation of our studies on the structure and biological role of triterpene oligoglycosides from holothurians [4,5,6,7,8,9,10], we have firstly investigated the ethanol (EtOH) extracts of the Stichopodidae-type sea cucumber *Stichopus variegates* Semper collected from the Hainan province in the South China sea which is used as a tonic in China [11]. Herein, we report the isolation and structure elucidation of the four new, unprecedented, non-sulfated triterpene glycosides, variegatusides C (**1**), D (**2**), E (**3**) and F (**4**). All isolated compounds revealed different antifungal activities.

## 2. Results and Discussion

**Figure 1:**
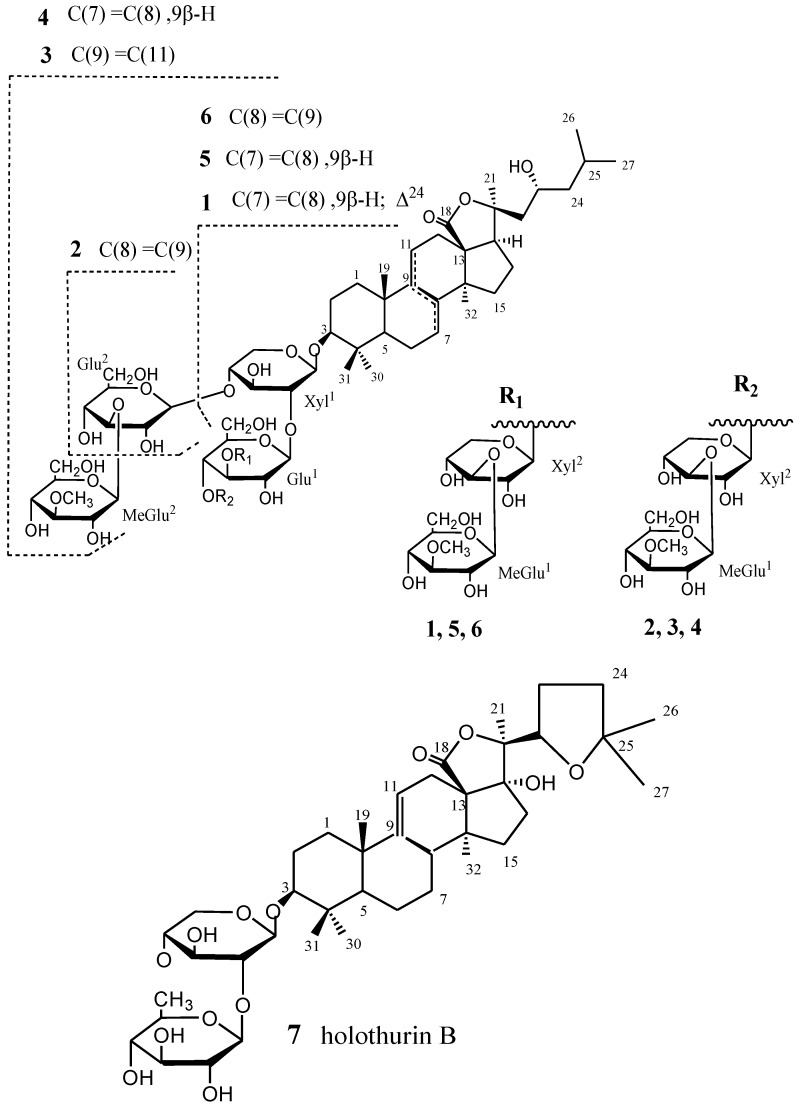
The structures of compounds (**1**–**7**).

The 70% ethanolic extract of *S. variegates* (15 kg, wet weight) was suspended in H_2_O and partitioned successively with petroleum ether and *n*-BuOH. The *n*-BuOH extract was subjected to silica gel and reversed-phase silica (Lichroprep RP-18, 40–63 μm). Final purification of individual compounds was achieved by reversed-phase HPLC on Zobax SB C-18 to give variegatusides C (**1**), D (**2**), E (**3**), F (**4**) and three known compounds **5**–**7** (Figure 1). Structures of the glycosides have been elucidated by extensive analysis (NMR and ESIMS) and chemical method. 

Variegatuside C (**1**), obtained as colorless amorphous powder, was positive to Liebermann-Burchard and Molish test. Its molecular formula was determined as C_53_H_84_O_22_ from the pseudomolecular ion peak at *m/z* 1071.5419 [M − H]^+^ in negative ion mode HRESIMS and at *m/z* 1095 [M + Na]^+^ in positive ion mode ESIMS and ^13^C NMR. The IR spectrum showed the presence of hydroxyl (3417 cm^−1^), carbonyl (1761 cm^−1^), and olefinic (1652 cm^−1^) groups.

The ^1^H NMR, ^13^C NMR and DEPT spectra of glycoside **1** showed the close aglycone with desacetylstichloroside B_1_ [12] and variegatuside A [13], differing only by the presence of an olefinic bond at 24(25). The ^1^H and ^13^C NMR spectral data of **1** (Table 1) suggested the presence of a triterpene aglycone with two olefinic bonds, one ester, and one hydroxyl group bonded to an oligosaccharide chain composed of four sugar units. Resonances for a 7(8)-double bond at δ_C_ 143.6 (C-8), 120.3 (C-7); δ_H_ 5.62 (*m*, H-7) and one 24(25)-double bond at δ_C_ 131.2 (C-25), 123.2 (C-24); δ_H_ 5.01 (*d*, *J* = 7.8 Hz, H-24) were present. The positions of two C=C bonds in 7,8 and 24,25 were corroborated by the HMBC correlations H-32/C-8, H-9/C-8, H-5/C-7, H-9/C-7, and H-27/C-25, H-24/C-25, H-27/C-24, H-23/C-24, H-22/C-24, respectively. The ^13^C NMR chemical shift inventory of **1** closely parallel that of frondoside A [14] except for signals assigned to C-22 (δ_C_ 48.9) and C-23 (δ_C_ 66.0) which are shifted downfield by δ 9.77 and 43.24, respectively, in agreement with the presence of an OH group at C-23. The side chain of aglycone is thus comparable to that of stichlorogenol [10], a C-23-hydroxy aglycone from the sea cucumber *Stichopus chloronotus* for which stereochemistry has been confirmed by X-ray crystallography [12]. Comparable signals in the ^13^C NMR spectrum (pyridine-*d*_5_) of the latter are observed at δ_C_ 47.62 and 65.70 [15], suggesting the 23S configuration in variegatuside C (**1**).

**Table 1:** ^1^H (600 MHz) and ^13^C NMR (150 MHz) Data and Selected HMBC Correlations for the Aglycone Moieties of **1** and **2 **(in C_5_D_5_N:D_2_O, 4:1) ^a^.

No.	1			2		
	δ_H_ mult. (*J* in Hz)	δ_C_	HMBC (H→C)	δ_H_ mult. (*J* in Hz)	δ_C_	HMBC (H→C)
1	1.20 m; 1.70 m	36.2, CH_2_		1.17 m; 1.72 m	36.2, CH_2_	
2	1.94 m; 2.21 m	27.2, CH_2_		1.90 m; 2.10 m	27.1, CH_2_	
3	3.31 dd (4.8, 9.6)	88.9, CH	4, 30, 31, 1 (Xyl^1^)	3.23 dd (4.8, 10.4)	88.7, CH	4, 30, 31, 1 (Xyl^1^)
4		39.8, C			39.6, C	
5	1.26 m	51.1, CH	3, 4, 10, 19, 30	1.13 m	51.1, CH	3, 4, 10, 19, 30, 31
6	1.65 m; 1.86 m	22.1, CH_2_		1.67 m	18.4, CH_2_	
7	5.62 m	120.3, CH	5, 6, 8	2.14 m; 2.20 m	27.1, CH_2_	5, 6, 8
8		143.6, C			131.1, C	
9	3.72 m	48.7, CH	8, 10, 11, 19		135.6, C	8, 10, 11, 19
10		37.2, C			37.1, C	
11	1.32 m; 1.46 m	23.3, CH_2_		2.22 m; 2.34 m	21.8, CH_2_	
12	2.05 m	33.2, CH_2_	9,11,13,18	2.12 m; 2.19 m	28.2, CH_2_	9,11,13,18
13		59.0, C			58.9, C	
14		51.1, C			49.0, C	
15	1.80 m	33.6, CH_2_		1.48 m; 1.60 m	33.2, CH_2_	
16	1.62 m; 1.74 m	28.2, CH_2_		1.96 m; 2.10 m	25.1, CH_2_	
17	2.48 m	52.7, CH	16, 20, 21	2.41 m	51.4, CH	16, 20, 21
18		177.6, C			177.4, C	
19	1.11 s	22.3, CH_3_	1, 5, 9, 10	1.26 s	19.2, CH_3_	1, 5, 9, 10
20		83.7, CH			84.0, C	
21	1.67 s	27.4, CH_3_	17, 20, 22	1.81s	28.5, CH_3_	17, 20, 22
22	1.64 m; 2.34 m	48.9, CH_2_		2.02 m, 2.15 m	47.9, CH_2_	
23	4.20 m	66.0, CH	24, 25	4.02 m	65.7, CH	24, 25
24	5.01 d (7.8 )	123.2,CH	23, 25, 26,27	1 34 m; 1.68 m	49.2, CH_2_	23, 25, 26, 27
25		131.2, C		2.04 m	24.7, CH	
26	0.96 d (6.6)	26.4, CH_3_	24, 25, 27	1.00 d (6.8)	23.8, CH_3_	24, 25, 27
27	1.30 d (6.0)	19.4, CH_3_	24, 25, 26	1.01 d (6.8)	22.1, CH_3_	24, 25, 26
30	1.10 s	16.7, CH_3_	3, 4, 5, 31	1.07s	16.5, CH_3_	3, 4, 5, 31
31	1.26 s	28.2, CH_3_	3, 4, 5, 30	1.23 s	28.0, CH_3_	3, 4, 5, 30
32	1.04 s	28.3, CH_3_	8, 13, 14, 15	1.05 s	25.4, CH_3_	8, 13, 14, 15

^a^ Assignments aided by DQFCOSY, TOCSY, HMQC, HMBC and NOESY experiments.

The presence of four monosaccharide units in the sugar chain of the glycoside **1** was deduced from its ^13^C NMR and DEPT spectra, which showed four anomeric carbons at 105.8, 105.9, 105.0, and 105.6 ppm, correlated by HMQC to their corresponding anomeric protons at 4.83 (d, *J* = 7.2 Hz), 5.28 (d, *J* = 7.8 Hz), 5.11 (d, *J* = 7.8 Hz), and 5.25 (d, *J* = 7.2 Hz) ppm (Table 1). The coupling constants of the anomeric protons were indicative in all cases of a β-configuration for the glycosidic bonds [16]. The monosaccharide units in **1** were identified as xylose, glucose, and 3-*O*-methylglucose in a 2:1:1 ratio by acidic hydrolysis with aqueous 2 mol/L trifluoroacetic acid and preparation of the corresponding standard aldonitrile peracetates (Sigma), which were analyzed by GC-MS. The NMR spectral data of the carbohydrate part of glycoside **1** were coincident with those of thelenotoside B from *Thelenota ananas* [17], indicating that these two glycosides contain the same carbohydrate chain.

**Figure 2:**
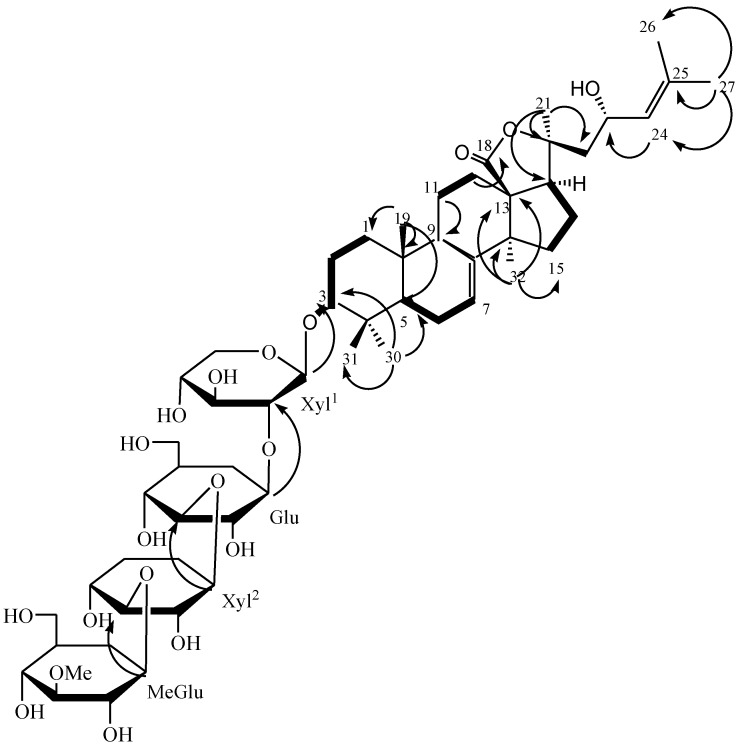
The ^1^H–^1^H COSY (bold lines) and selected HMBC (arrows) correlations for **1**.

The DQFCOSY experiment allowed the sequential assignment of most of the resonances of each sugar ring, starting from the easily distinguished signals due to anomeric protons. Complete assignment was achieved by a combination of DQFCOSY and TOCSY results. The HMQC experiment correlated all proton resonances with those of their corresponding carbons. These data (Table 2) indicated that the four sugar residues are in their pyranose forms. The locations of the interglycosidic linkages were deduced from the chemical shifts of Xyl^1^ C-2 (δ_C_ 83.7), Glu C-3 (δ_C_ 80.4) and Xyl^2^ C-3 (δ_C_ 87.6), which were downfield relative to shifts expected for the corresponding methyl glycopyranosides [16]. The sequence of the sugar residues in **1** was determined by analysis of HMBC correlations: Xyl^1^ H-1/C-3 of the aglycone, Glu H-1/Xyl^1^ C-2, Xyl^2^ H-1/ Glu C-3 and MeGlu H-1/Xyl^2^ C-3. This conclusion was also confirmed by the NOESY correlations as shown in Figure 2.

**Table 2:** ^1^H- (600 MHz) and ^13^C-NMR (150 MHz) Data and Key HMBC Correlations for the Sugar Moieties of **1** and **2** (in C_5_D_5_N:D_2_O, 4:1) ^a^.

No.	1			2		
	δ_H_ mult. (*J* in Hz)	δ_C_	HMBC (H→C)	δ_H_ mult. (*J* in Hz)	δ_C_	HMBC (H→C)
Xyl^1^						
1	4. 83 d (7.2)	105.8, CH	3 (Aglycone)	4.72 d (7.6)	104.9, CH	3 (Aglycone)
2	4.17 m	83.7, CH		4.08 m	82.9, CH	
3	4.10 m	78.0, CH		4.18 m	75.5, CH	
4	3.99 m	71.0, CH		3.95 m	77.4, CH	
5	3.70 m, 4.34 m	66.6, CH_2_		3.66 m; 4.40 m	63.6, CH_2_	
Glu^1^						
1	5.28 d (7.8)	105.9, CH	2 (Xyl^1^)	5.20 d (6.8)	105.6, CH	2 (Xyl^1^)
2	3.94 m	76.9, CH		4.02 m	75.6, CH	
3	4.41 m	80.4, CH		4.04 m	69.1, CH	
4	3.74 m	69.2, CH		4.32 m	80.5, CH	
5	4.21 m	78.4, CH		3.98 m	75.7, CH	
6	4.26 m; 4.44 m	62.2, CH_2_	4, 5 (Glu^1^)	4.20 m; 4.44 m	62.3, CH_2_	C-4, 5 (Glu^1^)
Xyl^2^						
1	5.11 d (7.8)	105.0, CH	3 (Glu^1^)	5.05 d (6.8)	105.2, CH	4 (Glu^1^)
2	3.83 m	73.8, CH		3.96 m	73.1, CH	
3	4.07 m	87.6, CH		4.02 m	87.7, CH	
4	4.17 m	70.6, CH		4.06 m	69.8, CH	
5	3.58 m; 4.16 m	66.8, CH_2_		3.55 m; 4.15m	66.5, CH_2_	
Meglu^1^						
1	5.25 d (7.2)	105.6, CH	3 (Xyl^2^)	5.18 d (7.6)	105.4, CH	C-3 (Xyl^2^)
2	4.05 m	76.8, CH		3.98 m	73.6, CH	
3	3.73 m	88.1, CH		3.68 m	87.8, CH	
4	4.14 m	75.2, CH		4.09 m	70.7, CH	3, 5 (Meglu^1^)
5	3.82 m	75.7, CH	3 (Meglu^1^)	3.82 m	76.6, CH	
6	4.42 m; 4.60 m	61.2, CH_2_		4.38 m; 4.54 m	61.3, CH_2_	
OMe	3.87 s	60.9, CH_3_	3 (Meglu^1^)	3.85 s	60.6, CH_3_	3 (Meglu^1^)
Glu^2^						
1				4.96 d (7.6)	102.9, CH	4 (Xyl^1^)
2				3.98 m	75.1, CH	
3				3.94 m	78.2, CH	
4				4.10 m	70.7, CH	5 (Glu^2^)
5				4.20 m	77.7, CH	
6				4.22 m; 4.42 m	62.3, CH_2_	
MeGlu^2^						
1						
2						
3						
4						
5						
6						
OMe						

^a^ Assignments aided by DQFCOSY, TOCSY, HMQC, HMBC and NOESY experiments.

All these data indicate that variegatuside C (1) is 3β-*O*-[3-*O*-methyl-β-d-glucopyranosyl-(1→3)-β-d-xylopyranosyl-(1→3)-β-d-glucopyranosyl-(1→2)-β-d-xylopyranosyl]-23(*S*)-hydroxylholosta-7,24-diene.

Variegatuside D (**2**) was isolated as a colorless amorphous powder. It had a molecular formula of C_59_H_96_O_27_ on the basis of pseudomolecular ion peaks at *m/z* 1259.6031 [M + Na]^+^ (calcd. 1259.6037) in positive ion mode HRESIMS and at *m/z* 1235 [M − H]^−^ in the negative ion mode ESIMS. The IR spectrum showed the presence of hydroxyl (3355 cm^−1^), carbonyl (1760 cm^−1^), and olefinic (1633 cm^−1^) groups.

The ^13^C NMR spectral data of the aglycone parts of the glycoside **2** (Table 1) were found to be identical to those of the aglycone of variegatuside B [13], which had been previously identified as 23(*S*)-hydroxylholosta-8(9)-ene-3β-ol. And in the downfield region of the ^13^C NMR spectrum of **2**, signals at δ_C_ 131.1 and 135.6 are present. Such signals were not characteristic for a 7(8)- or 9(11)-double bond. The DEPT and HMQC spectra of variegatuside D (**2**) indicated that these signals are signals of quaternary carbons belong to a 8(9)-double bond. This structure for the glycoside **2** was confirmed by the NMR spectra and ^1^H–^1^H COSY, HMBC, HMQC, TOCSY and NOESY.

The five β-monosaccharide units were identified as xylose, glucose, and 3-*O*-methyl glucose in a 2:2:1 ratio based on the ^1^H and ^13^C NMR spectra, which showed five anomeric carbons (δ_C_ 104.9, 105.6, 105.2, 105.4 and 102.9) and five corresponding anomeric protons (δ_H_ 4.72, 5.20, 5.05, 5.18 and 4.96) resonances with coupling constants (*J* values) of 6.8–7.8 Hz (Table 2) and by acidic hydrolysis with aqueous 2 mol/L trifluoroacetic acid followed by GC-MS analysis of the corresponding aldononitrile peracetates. The sequence of the sugar residues [3-*O*-methylglc (1→3)-xyl (1→4)-glu(1→2) [glu (1→4)]-xyl (1→3)-aglycone] in compound **2** was determined by careful analysis of the HMBC cross-peaks, δ 4.72/88.7 H-1 (Xyl^1^)/C-3 (aglycone), 5.20/82.9 H-1 (Glu^1^)/C-2 (Xyl^1^), 5.05/80.5 H-1 (Xyl^2^)/C-4 (Glu^1^), 5.18/87.7 H-1 (Meglu^1^)/C-3 (Xyl^2^), and 4.96/77.4 H-1(Glu^2^)/C-4(Xyl^1^).

Therefore, the structure of compound **2** was deduced as 3β*-O*-[3-*O*-methyl-β-d-glucopyranosyl-(1→3)-β-d-xylopyranosyl-(1→4)-β-d–glucopyranosyl-(1→2)[β-d-glucopyranosyl-(1→4)]-β*-*d-xylopyranosyl]-23(*S*)-hydroxylholosta-8(9)-ene.

Variegatuside E (**3**) obtained as a colorless amorphous powder and minor component, was positive to Liebermann-Burchard and Molish test. The molecular formula of variegatuside E (**3**) was determined as C_66_H_108_O_32_ by ^13^C NMR and ESIMS. The positive ion mode ESIMS showed an [M + Na]^+^ ion peak at *m/z* 1435 [M + Na]^+^ and at *m/z* 1411 [M − H]^−^ in the negative-ion mode ESIMS. The IR spectrum showed the presence of hydroxyl (3438 cm^−1^), carbonyl (1743 cm^−1^), and olefinic (1649 cm^−1^).

The NMR data of compound **3** (Table 3) suggested that the aglycone of **3 **was quite comparable to those of variegatuside A [13], differing only from the position of an olefinic bond at 9(11). Resonances for a 9(11)-double bond [δ_C_ 151.2 (C-9) and 111.0 (C-11); δ_H_ 5.60 (1H, brd, H-11)] were present. The position of C=C bond in 9, 11 was corroborated by the HMBC correlations Me-19/C-9, H-5/C-9, and H-2/C-11. This conclusion was confirmed from the TOCSY and ^1^H–^1^H COSY spectrum.

The presence of six monosaccharide units in the sugar chain of the glycoside **3** was deduced from its ^13^C NMR and DEPT spectra, which showed six anomeric carbons at δ_C_ 104.9, 105.0, 104.5, 103.1, 102.3 and 105.2 ppm, correlated by HMQC to δ_H_ 4.65 (d, *J* = 7.2 Hz), 5.11 (d, *J* = 7.8 Hz), 4.95 (d, *J* = 7.2 Hz), 5.26 (d, *J* = 7.8 Hz), 4.88 (d, *J* = 7.2 Hz), and 5.28 (d, *J* = 7.2 Hz) ppm (Table 4). The oligosaccharide chain of **3** was quite identical to that of desacetystichloroside B_1_ [12]. The monosaccharide units in **3** were identified as xylose, glucose, and 3-*O*-methylglucose in a 1:1:1 ratio by acidic hydrolysis with aqueous 2 mol/L trifluoroacetic acid followed by GC-MS analysis of the corresponding aldonitrile peracetates.

**Table 3:** ^1^H (600 MHz) and ^13^C NMR (150 MHz) Data and Selected HMBC Correlations for the Aglycone Moieties of **3** and **4 **(in C_5_D_5_N:D_2_O, 4:1) ^a^.

No.	3			4		
	δ_H_ mult. (*J* in Hz)	δ_C_	HMBC (H→C)	δ_H_ mult. (*J* in Hz)	δ_C_	HMBC (H→C)
1	1.21m; 1.67 m	36.1, CH_2_		1.47 m	36.3, CH_2_	
2	1.95 m	26.6, CH_2_		1.95 m	27.2, CH_2_	
3	3.24 dd (6.0,12.0)	88.5, CH	4, 30, 31, 1 (Xyl^1^)	3.27 dd (4.2, 11.4)	89.0, CH	4, 30, 31, 1 (Xyl^1^)
4		39.6, C			39.6, C	
5	0.90 m	52.6, CH	3, 4, 10, 19, 30, 31	1.01 m	48.1, CH	3, 4, 10, 19, 30
6	1.20 m; 1.40 m	20.8, CH_2_		1.97 m	23.1, CH_2_	
7	1.26 m	27.8, CH_2_		5.69 m	120.0, CH	5, 6, 8
8	3.24 m	39.7, CH			147.1, C	
9		151.2, C	8, 10, 11, 19	3.44 m	47.6, CH	8, 10, 11, 19
10		39.2, C			35.6, C	
11	5.60 brd (4.8)	111.0, CH		1.80 m	23.4, CH_2_	
12	2.04 m	29.2, CH_2_	9, 11, 13, 18	1.90 m; 2.10 m	30.6, CH_2_	9, 11, 13, 18
13		58.0, C			58.7, C	
14		47.2, C			51.4, C	
15	1.33 m; 1.67 m	35.3, CH_2_		1.70 m; 1.82 m	34.4, CH_2_	
16	1.90 m	24.5, CH_2_		1.88 m; 2.10 m	24.7, CH_2_	
17	2.50 dd (4.8, 10.8)	51.7, CH		2.44 dd (4.8, 9.6)	53.8, CH	16, 20, 21
18		178.9, C			180.7, C	
19	1.38 s	21.7, CH_3_	1, 5, 9, 10	1.21 s	28.9, CH_3_	1, 5, 9, 10
20		84.5, C			85.0, C	
21	1.80 s	28.3, CH_3_	17, 20, 22	1.83 s	28.1, CH_3_	17, 20, 22
22	2.02 m; 2.19 m	47.0; CH_2_		2.02 m; 2.13 m	47.5, CH_2_	
23	4.08 m	65.0, CH	25	4.01 m	65.5, CH	24, 25
24	1.26 m; 1.63 m	48.8, CH_2_	23, 25, 26, 27	1.28 m; 1.64 m	49.3, CH_2_	23, 25, 26, 27
25	2.03 m	24.3, CH		1.91 m	25.3, CH	
26	1.02 s	23.5, CH_3_	24, 25, 27	1.00 d (6.0)	22.2, CH_3_	24, 25, 27
27	0.98 s	21.9, CH_3_	24, 25, 26	0.96 d (6.6)	23.8, CH_3_	24, 25, 26
30	1.07 s	16.4, CH_3_	3, 4, 5, 31	1.10 s	17.5, CH_3_	3, 4, 5, 31
31	1.22 s	27.8, CH_3_	3, 4, 5, 30	1.12 s	30.0, CH_3_	3, 4, 5, 30
32	0.87 s	19.6, CH_3_	8, 13, 14, 15	1.11 s	31.0, CH_3_	8, 13, 14, 15

^a^ Assignments aided by DQFCOSY, TOCSY, HMQC, HMBC and NOESY experiments.

The interglycosidic linkages in the hexasaccharide chain of 3 and its bonding to aglycone were confirmed by NOESY experiments that showed cross-peaks between H-1 of the first xylose residue and H-3 of the aglycone, between H-1 of glucose and H-2 of the first xylose residue, between H-1 of the second xylose residue and H-4 of the first glucose residue, between H-1 of the first 3-*O*-methylglucose and H-3 of the second xylose residue, between H-1 of the second glucose and H-4 of the first xylose residue, and between H-1 of the second 3-*O*-methylglucose and H-3 of the second glucose residue. Therefore, the structure of variegatuside E (3) is 3β-*O*-[3-*O*-methyl-β-d-glucopyranosyl-(1→3)-β-d-xylopyranosyl-(1→4)-β-d-glucopyranosyl-(1→2)[3-*O*-methyl-β-d-glucopyranosyl-(1→3)-β-d-glucopyranosyl-(1→4)]–β*-*d-xylopyranosyl]-23(*S*)-hydroxylholosta-9(11)-ene.

**Table 4:** ^1^H- (600 MHz) and ^13^C-NMR (150 MHz) Data and Key HMBC Correlations for the Sugar Moieties of 3 and 4 (in C_5_D_5_N:D_2_O, 4:1) ^a^.

No.	3			4		
	δ_H_ mult. (*J* in Hz)	δ_C_	HMBC (H→C)	δ_H_ mult. (*J* in Hz)	δ_C_	HMBC (H→C)
Xyl^1^						
1	4.65 d (7.2)	104.9, CH	3 (Aglycone)	4.77 d (7.2)	105.3, CH	3 (Aglycone)
2	4.06 m	82.0, CH		4.11 m	83.2, CH	
3	4.12 m	75.3, CH		4.18 m	75.7, CH	
4	3.95 m	77.2, CH		4.00 m	76.8, CH	
5	3.60 m; 4.12 m	64.7, CH_2_		3.64 m; 4.40 m	64.2, CH_2_	
Glu^1^						
1	5.11 d (7.8)	105.0, CH	2 (Xyl^1^)	5.23 d (7.8)	105.7, CH	2 (Xyl^1^)
2	3.96 m	73.2, CH		3.98 m	73.2, CH	
3	3.99 m	68.8, CH		4.06 m	69.2, CH	
4	4.20 m	80.2, CH		4.38 m	80.3, CH	
5	4.18 m	75.4, CH		4.22 m	75.4, CH	
6	4.11 m; 4.43 m	61.9, CH_2_	4, 5 (Glu^1^)	4.26 m; 4.42 m	62.1, CH_2_	4, 5 (Glu^1^)
Xyl^2^						
1	4.95 d (7.2)	104.5, CH	4 (Glu^1^)	5.11 d (7.8)	105.0, CH	4 (Glu^1^)
2	3.95 m	73.5, CH		4.02 m	73.1, CH	
3	4.12 m	87.4, CH	1 (Meglu^1^)	4.10 m	87.0, CH	1 (Meglu^1^)
4	4.00 m	69.3, CH		4.04 m	68.6, CH	
5	3.56 m; 4.10 m	66.1, CH_2_		3.56 m; 4.19 m	66.6, CH_2_	
Meglu^1^						
1	5.26 d (7.8)	103.1, CH	3 (Xyl^2^)	5.03 d (7.2)	105.8, CH	3 (Xyl^2^)
2	4.01 m	76.2, CH	3 (Meglu^1^)	3.98 m	75.2, CH	3 (Meglu^1^)
3	3.73 m	87.6, CH		3.76 m	88.1, CH	
4	3.95 m	70.4, CH		4.13 m	70.6, CH	
5	3.90 m	77.9, CH	4 (Meglu^1^)	3.95 m	78.4, CH	4 (Meglu^1^)
6	4.10 m; 4.32 m	61.9, CH_2_		4.26 m; 4.46 m	62.2, CH_2_	
OMe	3.87 s	60.5, CH_3_	3 (Meglu^1^)	3.86 s	60.8, CH_3_	3 (Meglu^1^)
Glu^2^						
1	4.88 d (7.2)	102.3, CH	4 (Xyl^1^)	4.98 d (7.2)	102.9, CH	4 (Xyl^1^)
2	3.93 m	75.9, CH		4.00 m	73.7, CH	
3	4.08 m	87.0, CH	1 (Meglu^2^)	4.06 m	87.6, CH	1 (Meglu^2^)
4	4.03 m	70.4, CH		4.08 m	69.7, CH	
5	3.94 m	77.6, CH		3.94 m	78.4, CH	
6	4.10 m; 4.40 m	61.0, CH_2_		4.24 m; 4.48 m	62.2, CH_2_	
MeGlu^2^						
1	5.28 d (7.2)	105.2, CH	3 (Glu^2^)	5.25 d (7.8)	105.6, CH	3 (Glu^2^)
2	4.02 m	76.1, CH		3.99 m	75.2, CH	
3	3.72 m	87.5, CH	2, -4 (Meglu^2^,OMe)	3.74 m	88.1, CH	2, 4 (Meglu^2^,OMe)
4	4.05 m	71.0, CH		4.15 m	70.6, CH	
5	3.80 m	78.0, CH		3.96 m	77.5, CH	
6	4.10 m; 4.44 m	61.9, CH_2_		4.20 m; 4.41 m	61.1, CH_2_	
OMe	3.86 s	60.5, CH_3_	3 (Meglu^2^)	3.85 s	60.8, CH_3_	3 (Meglu^2^)

^a^ Assignments aided by DQFCOSY, TOCSY, HMQC, HMBC and NOESY experiments.

Variegatuside F (**4**), white powder, the molecular formula was established as C_66_H_108_O_32_ from pseudomolecular ion peak at *m/z* 1411.6458 [M − H]^+^ in negative ion mode HRESIMS and at *m/z* 1435 [M + Na]^+^ in the positive ion mode ESIMS and ^13^C NMR. The IR spectrum showed the presence of hydroxyl (3418 cm^−1^), carbonyl (1750 cm^−1^), and olefinic (1635 cm^−1^) groups.

The analysis of ^13^C NMR spectral data of the aglycone moiety of variegatuside F (**4**) showed the presence of the signal at δ_C_ 180.7 (C-18) characteristic for the 18(20)-lactone. The signals of carbons C-22~C-27 were coincident with the corresponding signals in the ^13^C NMR spectrum of variegatuside A [13]. This indicated its side chains have a hydroxyl group at δ_C_ 65.5 (C-23) (Table 3). Glycoside 4 is a native compound from the sea cucumber *S. variegates* Semper which has been reported as desacetylstichloroside B_1_ [12]. The ^1^H and ^13^C NMR spectral data of 4 (Table 3) suggested the presence of a triterpene aglycone with an olefinic bond, one ester, and one hydroxyl group bonded to an oligosacchaide chain. Resonances for a 7(8)-double bond at δ_C_ 147.1 (C-8), 120.0 (C-7); δ_H_ 5.69 (m, H-7) were present. The position of the C=C bonds in 7, 8 was corroborated by the HMBC correlations H-32/C-8, H-9/C-8, H-5/C-7, H-9/C-7, respectively. The aglycone structure proposed for variegatuside F (**4**) containing an 7(8)-double bond and a 23-hydroxyl group is confirmed by ^1^H–^1^H COSY, HMQC, TOCSY, NOESY, and HMBC spectral data (Table 3).

Comparison of the NMR spectra of carbohydrate moieties of variegatuside E (**3**) and variegatuside F (**4**) (Table 4) shows coincidence of all monosaccharide residue signals. This indicates the identity of the sugar parts of two glycosides. The sequence of the sugar residues in **4** was determined by analysis of HMBC correlations: Xyl^1^ H-1/C-3 of the aglycone, Glu^1^ H-1/Xyl^1^ C-2, Xyl^2^ H-1/Glu^1^ C-3, MeGlu^1^ H-1/Xyl^2^ C-3, Glu^2^ H-1/Xyl^1^ C-4 and MeGlu^2^ H-1/Glu^2^ C-3. This conclusion was also confirmed by the NOESY correlations.

Therefore, variegatuside F (**4**) is 3β-*O*-[3-*O*-methyl-β-d-glucopyranosyl-(1→3)-β-d-xylopyranosyl-(1→4)-β-d-glucopyranosyl-(1→2)[3-*O*-methyl-β-d-glucopyranosyl-(1→3)-β-d-glucopyranosyl-(1→4)]-β*-*d-xylopyranosyl]-23(*S*)-hydroxylholosta-7(8)-ene. Compound **4** is a native compound from the sea cucumber *S. variegates* Semper which has been reported as desacetylstichloroside B_1_.

**Table 5:** Antifugal effects of triterpene glycosides (MIC_80_: μg/mL).

	*C. albicans*	*C. pseudotropicalis*	*C. neoformans*	*C. parapsilosis*	M. *gypseum*	*C. tropicalis*
**1**	12.5	25	50	25	12.5	12.5
**2**	3.4	3.4	6.8	3.4	3.4	13.6
**3**	25	12.5	12.5	12.5	12.5	12.5
**4**	25	12.5	25	12.5	12.5	12.5
**5**	25	25	12.5	12.5	12.5	12.5
**6**	100	125	50	25	25	>125
**7**	18.8	18.8	9.4	>125	>125	37.6
FCZ ^a^	1	4	4	1	>64	1
ICZ ^a^	0.25	0.125	0.5	0.125	0.125	0.5
KCZ ^a^	1	0.125	0.5	0.125	0.125	0.125

^a^ Positive antifungal activity control; MIC80: the minimum concentration to inhibit ≤80% growth for **1**–**7** against six fungi strains.

Some triterpene glycosides hitherto isolated from sea cucumber exhibited significant antifungal activity [15]. Glycosides **1**–**7** exhibited selective antifungal activities against six strains while **2**, **3** had significant growth inhibitory activities against six strains (Table 5). These facts suggest that the Δ^25^ terminal double bond may increase the activity. The component of the carbohydrate chain seems play an important role whereas the position of trisubstituted double bond in aglycone moiety (Δ^7^, Δ^8^ or Δ^9(11)^) contributes little to the bioactivity. Therefore, more extensive studies are needed before a clear structure-activity relationship can be reached.

## 3. Experimental Section

### 3.1. General Experimental Procedures

Melting points were determined on an XT5-XMT apparatus. Optical rotations were measured on a Perkin-Elmer-341 polarimeter (PerkinElmer). IR spectra were recorded on a Bruker Vector-22 infrared spectrometer (Bruker). NMR spectra were obtained from Varian Inova-600 spectrometer with standard pulse sequence. ESI- and HR-ESI-MS were acquired on Micromass Quattro mass spectrometer (Varian). GC-MS were performed on a Finnigan Voyager apparatus (Finnigan Voyager, San Jose, CA, USA) using a DB-5 column (30 m × 0.25 mm i.d., 0.25 μm). HPLC was carried out on an Agilent 1100 liquid chromatograph (Agilent Technologies, Palo Alto, CA, America) equipped with a refractive index detector using a Zorbax 300 SB-C_18_ column (250 × 9.4 mm i.d.) (Agilent). Column chromatographic separations were performed on silica gel H (200–300 mesh, 10–40 μm, Qingdao Marine Chemical Inc.; Qingdao, China), Lobar Lichroprep RP-C_18_ (40–63 μm; Merck) and Sephadex LH-20 (Pharmacia). Fractions were monitored by TLC on precoated silica gel HSGF_254_ plates (CHCl_3_-EtOAc-MeOH-H_2_O, 4:4:2.5:0.5) or RP-C_18_ (MeOH-H_2_O, 1:1), (Qingdao Marine Chemical Inc., Qingdao, China) and spots were visualized by heating Si gel plates (Qingdao Marine Chemical Inc., Qingdao, China) sprayed with 15% H_2_SO_4_ in EtOH.

### 3.2. Animal Material

Specimens of *S. variegates* were collected in the South China Sea (Hainan, China) in October 2004, and authenticated by Yu-Lin Liao (Institute of Oceanology, Chinese Academy of Science, Qingdao, China). A voucher specimen (No.HYSC-200410) was deposited in the Research Center for Marine Drugs, School of Pharmacy, Second Military Medical University (Shanghai, China).

### 3.3. Extraction and Isolation

The sea cucumber (15 kg, wet weight) were crumbled and extracted at room temperature three times with 70% ethanol (20 L, 7 days for each extraction). The combined extracts were concentrated to leave a rufous residue, which was suspended in H_2_O and then partitioned successively with petroleum ether and *n*-BuOH. The *n*-BuOH fraction (17.6 g) was chromatographed on silica gel eluting with CHCl_3_-MeOH-H_2_O (8.2:1.8:1 to 6.5:3.5:1, lower phase) gradient to give three fractions (A–C) based on thin-layer chromatography (TLC) analysis. Fractions B and C mainly contained triterpene glycosides. Fr. B (2.1g) was subjected to reversed-phase silica MPLC (Lichroprep RP-C_18_, 40–63 μm,) eluting with an aq. MeOH (30%–70%) gradient to give 5 sub-fractions, and the Fr. B3 (165 mg) was purified by HPLC (Zorbax 300 SB-C_18_, 5 μm; 250 × 9.4 mm i.d.; 65% aq. MeOH, 1.5 mL/min) to yield glycoside **1** (9 mg, *t*_R_ 43.3 min), **5 **(19.6 mg; *t*_R_ 36.2 min) and **6 **(51 mg; *t*_R_ 60.1 min). Fr. C (910 mg) was subjected to size exclusion chromatography on a Sephadex LH-20 column equilibrated with MeOH:H_2_O (4:6) to give 2 sub-fractions. The Fr. C1 (210 mg) was purified by HPLC (using MeOH-H_2_O 76:24 as the mobile phase at flow rate of 1.5 mL/min) to yield pure glycosides **4** (9.5 mg; *t*_R_ 28.4 min), **2** (16.8 mg; *t*_R_ 33.5 min) and **3 **(3.6 mg; *t*_R_ 36.8 min).

#### 3.3.1. Variegateside C (**1**)

Colorless amorphous powder; m.p. 235–237 °C; 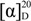: −16.7 (c 0.5, pyridine); IR (KBr) ν_max_ = 3417, 1761 and 1652 cm^−1^; ^1^H and ^13^C NMR data, see Table 1 and Table 2; ESIMS *m/z* 1095 [M + Na]^+^, *m/z* 1071 [M − H]^−^; HRESIMS *m/z* 1071.5419 [M − H]^−^ (calcd. for C_53_H_83_O_22_^−^, 1071.5431). 

#### 3.3.2. Variegateside D (**2**)

Colorless amorphous powder; m.p. 200–203 °C; 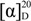: −26.4 (c 0.55, pyridine); IR (KBr) ν_max_ = 3355, 1760 and 1633 cm^−1^; ^1^H and ^13^C NMR data, see Table 1 and Table 2; ESIMS *m/z* 1259 [M + Na]^+^, *m/z* 1235 [M − H]^−^; HRESIMS *m/z* 1259.6031 [M + Na]^+^ (calcd. for C_59_H_9__6_O_27_Na, 1259.6037).

#### 3.3.3. Variegateside E (**3**)

Colorless amorphous powder; m.p. 250–251 °C; 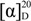: −32 (c 0.5, pyridine); IR (KBr) ν_max_ = 3438, 1743 and 1649 cm^−1^; ^1^H and ^13^C NMR data, see Table 3 and Table 4; ESIMS *m/z* 1435 [M + Na]^+^, *m/z* 1411 [M − H]^−^; HRESIMS *m/z* 1411.6709 [M − H]^−^ (calcd. for C_66_H_107_O_32_^−^, 1411.6714).

#### 3.3.4. Variegateside F (**4**)

Colorless amorphous powder; m.p. 210–212 °C; 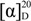: −34 (c 0.5, pyridine); IR (KBr) ν_max_ = 3418, 1750 and 1635 cm^−1^; ^1^H and ^13^C NMR data, see Table 3 and Table 4; ESIMS *m/z* 1435 [M + Na]^+^, *m/z* 1411 [M − H]^−^; HRESIMS *m/z* 1411.6458 [M − H]^−^ (calcd. for C_66_H_107_O_32_^−^, 1411.6465).

### 3.4. Acid Hydrolysis of Compounds **1**–**4**

Each glycoside (1 mg) was heated in an ampule with 2 mol/L trifluoroacetic acid (1 mL) at 120 °C for 2h. The reaction mixture was evaporated to dryness, and the residue was partitioned between CH_2_Cl_2_ and H_2_O. The aqueous phase was concentrated under reduced pressure. Then 1 mL of pyridine and 2 mg of NH_2_OH·HCl were added to the dried residue, and the mixture was heated at 90 °C for 30 min. After the reaction mixtures were cooled, Ac_2_O (0.8 mL) was added and the mixtures were heated at 90 °C for 1 h. The reaction mixtures were evaporated under reduced pressure, and the resulting aldononitrile peracetates were analyzed by GC-MS using standard aldononitrile peracetates (Sigma) as reference samples. d-xylose, d-glucose, 3-*O*-methyl-d-glucose were identified for variegateside C (**1**) in a 2:1:1 ratio, and variegateside D (**2**) in a 2:2:1 ratio, and in a 1:1:1 ratio for variegatesides E (**3**) and F (**4**).

### 3.5. Bioassay

The antifungal activities of the compounds **1**–**7** were tested against six strains (provided by the Changhai hospital, the Second Military Medical University, China): *Candida albicans* (ATCC76615), *Cryptococcus neoformans* (ATCC32609), *Microsporum gypseum* (ATCC31388), *Candida pseudotropicalis* (Clinic Strains), *Candida parapsilosis* (Clinic Strains) and *Candida tropicalis* (Clinic Strains). The antifungal activity data were evaluated by measuring optical density (OD) at 630 nm using Automatic Microplate Reader [18]. The drug MIC_80_ was defined as the first well with an approximate 80% reduction in growth compared to the growth of the drug-free well. The data represented the means of three independent experiments in which each compound concentration was tested in three replicate wells. Itraconazole (ICZ), Ketoconazole (KCZ), and Fluconazole (FCZ) were used as positive controls (Table 5).

## 4. Conclusions

This work represents the first study on the glycosidic contents of this South China sea cucumber *S. variegates*, a traditional tonic in China, led to the isolation of four holostane-type triterpene glycosides, together with three known triterpene glycosides. Variegatuside C–F (**1**–**4**) are new triterpene glycosides, which contain 23 (*S*)-hydroxyl in the side chain of the glycoside and non-sulphate in the sugar chain. These glycosides have Δ^7(8)^ and Δ9^(11)^, especially compounds **2 **and **6 **contain a rare 8(9)-double bond in both aglycone structures. All of these demonstrate an example of the chemical diversity from sea cucumber.

*In vitro* assays on the compounds indicated their potential as antifungal lead compounds. The structural novelty and biological activity of the secondary metabolites isolated from *S. variegates* Semper taxonomic group suggest that the sea cucumber has been overlooked in the search for new bioactive compounds, could potentially provide an interesting source of antifungal natural products and may warrant further biomedical investigation.
